# Evolutionarily Conserved Interaction between the Phosphoproteins and X Proteins of Bornaviruses from Different Vertebrate Species

**DOI:** 10.1371/journal.pone.0051161

**Published:** 2012-12-07

**Authors:** Kan Fujino, Masayuki Horie, Tomoyuki Honda, Shoko Nakamura, Yusuke Matsumoto, Ivo M. B. Francischetti, Keizo Tomonaga

**Affiliations:** 1 Department of Viral Oncology, Institute for Virus Research, Kyoto University, Kyoto, Japan; 2 Section of Vector Biology, Laboratory of Malaria and Vector Research National Institutes of Allergy and Infectious Diseases, National Institutes of Health, Bethesda, Maryland, United States of America; Mayo Clinic, United States of America

## Abstract

Bornavirus, a non-segmented, negative-strand RNA viruses, is currently classified into several genetically distinct genotypes, such as Borna disease virus (BDV) and avian bornaviruses (ABVs). Recent studies revealed that bornavirus genotypes show unique sequence variability in the putative 5′ untranslated region (5′ UTR) of X/P mRNA, a bicistronic mRNA for the X protein and phosphoprotein (P). In this study, to understand the evolutionary relationship among the bornavirus genotypes, we investigated the functional interaction between the X and P proteins of four bornavirus genotypes, BDV, ABV genotype 4 and 5 and reptile bornavirus (RBV), the putative 5′ UTRs of which exhibit variation in the length. Immunofluorescence and immunoprecipitation analyses using mammalian and avian cell lines revealed that the X proteins of bornaviruses conserve the ability to facilitate the export of P from the nucleus to the cytoplasm via interaction with P. Furthermore, we showed that inter-genotypic interactions may occur between X and P among the genotypes, except for X of RBV. In addition, a BDV minireplicon assay demonstrated that the X and P proteins of ABVs, but not RBV, can affect the polymerase activity of BDV. This study demonstrates that bornaviruses may have conserved the fundamental function of a regulatory protein during their evolution, whereas RBV has evolved distinctly from the other bornavirus genotypes.

## Introduction

Bornaviruses are non-segmented, negative-strand RNA viruses belonging to the order Mononegavirales and are characterized by highly neurotropic and noncytopathic infections [1], [2]. Borna disease virus (BDV), the prototype of the Bornaviridae family, infects a wide variety of mammalian species and causes a meningoencephalitis in naturally infected horses and sheep [3], [4]. Different isolates of BDV show high genetic conservation [5]–[7] and, until very recently, it was considered that BDV is the only member of the family Bornaviridae. However, recent studies have revealed that genetically divergent bornaviruses infect psittacine birds suffering from proventricular dilatation disease (PDD), a fatal disease characterized by a lymphocytic, plasmacytic inflammatory infiltrate of central and peripheral nervous tissues [8], [9]. These newly identified bornaviruses, avian bornavirus (ABV), have been confirmed to be a causative agent of PDD and also seem to infect in non-psittacine species, such as canaries (*Serinus canaria*) and Canada geese (*Branta canadensis*) [10], [11]. In addition, we recently detected sequences with significant sequence homology with the BDV nucleoprotein (N), X, and phosphoprotein (P) genes in a cDNA library derived from a Bitis gabonica (*Gaboon viper*) venom gland [12]. Because the genome DNA of Bitis gabonica seemed to not contain such BDV-like sequences, we have determined that the sequences are derived from an exogenous reptile bornavirus (RBV).

The heterogeneity of ABV isolates appears to be significantly higher than that of BDV and, to date, at least nine genotypes have been identified by phylogenetic analyses [8]–[11], [13], [14]. Furthermore, intriguingly, some genotypes of ABV seem to be more closely related genetically to BDV than other ABV [15]. Although infectious isolates have not yet been derived from many ABV genotypes, the comparison of the biological characteristics among the genotypes, including BDV and RBV, could provide a better understanding of the evolution, alteration of host range and the inter-vertebrate transmission of bornaviruses.

**Figure 1:**
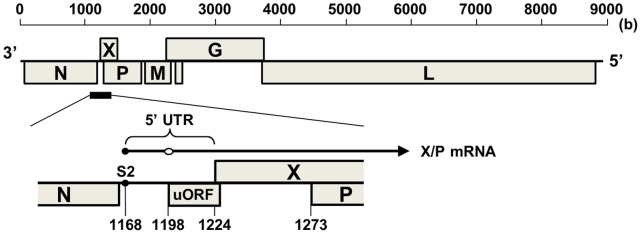
Schematic representation of BDV genome. An illustration of the genome organization of BDV is shown at the top. The genome region corresponding to the 5′ UTR of X/P mRNA is enlarged in the center. The arrow indicates a schematic structure of X/P mRNA. The open circle on X/P mRNA indicates the region of a predicted stem-loop structure. The regions of transcription start signal for X/P mRNA (S2) and uORF are shown. The number indicates the nucleotide position of the BDV genome (strain huP2br: Accession number AB258389).

Sequence analyses of non-mammalian bornaviruses revealed an interesting feature in the sequence between the N and X genes, which contains the region corresponding to the 5′ untranslated region (5′ UTR) of BDV X/P mRNA expressing both the X and P proteins (Figure 1). This region in ABV genotypes 2 and 4 (ABV2 and ABV4) lacks 22 nucleotides (nt) found in BDV isolates. Furthermore, we showed that RBV also contains a 21 nt deletion in the corresponding region [12]. On the other hand, it has been shown recently that ABV from Canada geese (ABV_CG_) has an almost full-length 5′ UTR in this region, similar to BDV [15]. This suggests that ABV_CG_ is much more closely related to BDV evolutionarily than are ABV2/4 and RBV. In a previous study, we have shown that the 5′ UTR of BDV X/P mRNA harbors regulatory sequences, such as a predicted stem-loop structure and a short upstream ORF (uORF) (Figure 1), that control the translation of the X protein [16]. The sequence variability in the 5′ UTR of these genotypes, therefore, may account for differences in the translation efficiency of X. In addition, BDV X is considered to regulate the viral polymerase activity by controlling the intranuclear amount of P through the direct interaction with P [16], [17]. These observations suggest that comparison of the function of the X and P proteins among various genotypes may provide interesting insights into the evolutionary relationship of bornaviruses.

In this study, we investigated the functional interaction between X and P among various vertebrate bornaviruses, which differ in the length of the putative 5′ UTR of X/P mRNA [12], [15], [18]. We show here conservation of the ability of the X protein of vertebrate bornaviruses to facilitate export of P from the nucleus to the cytoplasm via its interaction with P. Furthermore, we show that inter-genotypic interactions may occur between X and P, with the exception of the X protein of RBV. In addition, a BDV minireplicon assay revealed that the X proteins of ABVs, but not RBV, can inhibit the polymerase activity of BDV. Our results suggest that although RBV may have evolved the X protein in a genotype- and/or host-specific manner, the fundamental function of the X protein as a regulator of the intranuclear level of P has been preserved among bornaviruses throughout their evolution.

## Materials and Methods

### Cells

The OL cell line [19], derived from a human oligodendroglioma, and BDV-infected OL cells were cultured in Dulbecco’s modified Eagle’s medium containing 5% fetal bovine serum. Human HEK-293T cells and QT6 cells (American Type Culture Collection, CRL-1708), derived from quail were maintained in Dulbecco’s modified Eagle’s medium containing 10% fatal bovine serum. Cells were cultured at 37°C under 5% CO_2_.

### Plasmid Construction

To generate the eukaryotic expression plasmids, PCR amplified bornavirus X and P genes were cloned into the plasmid pcDNA3 (Invitrogen). The BDV X and P genes were amplified from cDNA from BDV-infected OL cells. The X gene primer included a Flag tag sequence and the P gene vector contained a HA tag sequence. Then, each X protein was expressed as a Flag fusion protein and each P protein was expressed as an HA fusion protein. Nucleotide sequences of the recombinant constructs were confirmed by DNA sequencing.

### Immunoprecipitation Assays

The 293T cells were seeded in 10 cm plates. One day after seeding, cells were transfected with Flag-tagged bornavirus X and/or HA-tagged bornavirus P plasmids using Lipofectamine 2000. At 24 h posttransfection, the media were removed from the plates by aspiration and the 293T cells were washed with PBS. Cells were then scraped with 1 ml PBS. After centrifugation (2,500 rpm, 1 min), the PBS was aspirated and the cells were lysed using lysis buffer (20 mM Tris-HCl pH 7.4, 150 mM NaCl, 1% Triton-X100, 1 mM EDTA, protease inhibitor). To homogenize, the cell lysates were sonicated and rotated for 30 min. After centrifugation (15,000 rpm, 20 min), the supernatants were incubated with 40 µl of pre-equilibrated anti-HA resin (Sigma-Aldrich) overnight with rotation. After incubation, beads were collected by centrifugation at 12,000 rpm for 1 min and washed three times with 1 ml of lysis buffer. The proteins immunoprecipitated with anti-HA resin were detected by western blotting. All methods used during the harvesting procedure were performed at 4°C. Western blot analysis was performed using standard techniques and 15% SDS polyacrylamide gel electrophoresis (PAGE). The rabbit anti-Flag antibody (Sigma-Aldrich) was diluted 1∶1,000, the rabbit anti-HA antibody (Santa Cruz) was diluted 1∶1,000 in 5% low-fat milk powder in PBS or Can Get Signal (TOYOBO) and incubated with membranes overnight at 4°C. After washing the samples three times for 10 min with PBS-0.1% Tween-20, antibodies were detected using horseradish peroxidase-coupled goat anti-rabbit or anti-mouse antibodies (Jackson ImmunoResearch) diluted 1∶5,000 in 5% low-fat milk powder in PBS or Can Get Signal, and visualization was performed using ECL Plus Western Blot Detection Reagents (GE Healthcare) according to the manufacturer’s instructions.

### Indirect Immunofluorescence Assays

OL cells or QT6 cells were seeded onto 8-well chamber slides. One day after seeding, the cells were transfected with Flag-tagged bornavirus X and/or HA-tagged P plasmids using Lipofectamine 2000. The next day, the cells were fixed for 15 min in 4% paraformaldehyde, permeabilized by incubation for 5 min in PBS containing 0.4% Triton X-100 and were treated with 1% bovine serum albumin. After reaction with mouse anti-HA antibody (Roche) and/or rabbit anti-Flag antibody (Sigma-Aldrich), the cells were stained with anti-rabbit Alexa488 (Invitrogen) and/or anti-mouse Alexa555 (Invitrogen) as the secondary antibody. First and second antibody reactions were performed for 1 h at 37°C. Fluorescence was detected using a confocal laser-scanning microscope.

### Minireplicon Assay

Minireplicon assays were carried out according to Yanai *et al*. (2006). Briefly, 293T cells were seeded in 12-well plates and transfected with expression plasmids of BDV N, P, RNA-dependent RNA polymerase (L) and Pol II-driven minigenome plasmids, with or without the bornavirus X expression plasmid, using Lipofectamine 2000 (Invitrogen). 48 h later, the cells were lysed and cell lysates were prepared for CAT assays. CAT activity was quantified with a CAT ELISA (Roche) according to the manufacturer’s directions.

## Results

### Sequence Comparison of the X and P Genes of Mammalian, Avian and Reptile Bornaviruses

**Figure 2:**
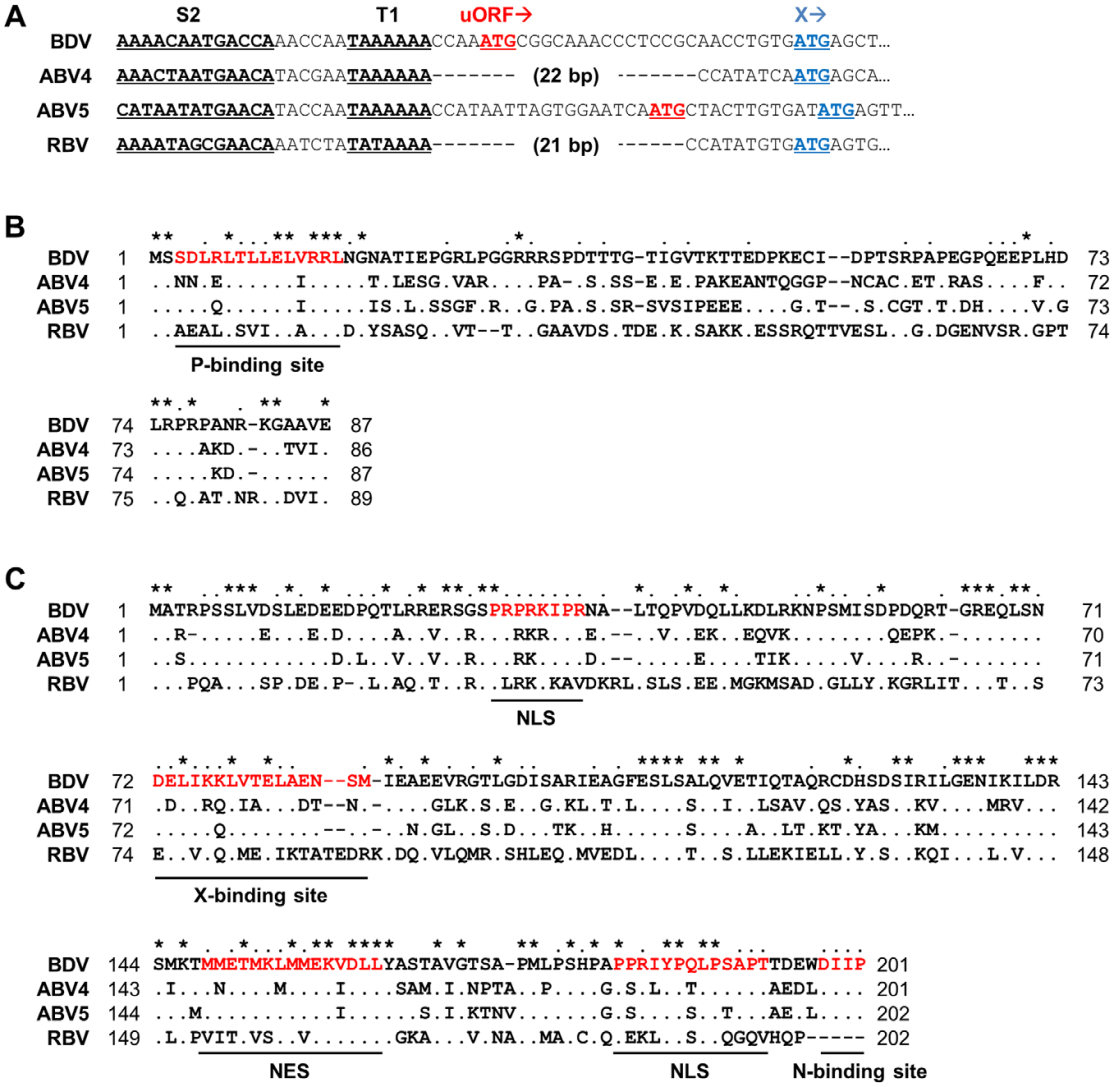
Sequence comparisons among bornavirus genotypes. (A) Nucleotide sequence alignment of the region between the N and X ORFs. The stop and start codons of the BDV N and X genes, respectively, are shown. The start codon of the BDV uORF and the proposed uORF of ABV5 are indicated in red. The S2 transcription initiation and T1 termination sites of BDV and their corresponding sequences in non-mammalian bornaviruses are underlined. (B and C) Amino acid sequence alignments of X (B) and P (C) among bornavirus genotypes. Amino acids identical to the BDV sequences are indicated by dots. Gaps are indicated by dashes. Sequences identical in four and three genotypes are indicated by asterisks and dots, respectively, above the sequences. The regions of NLS, NES and putative binding sites for each protein are underlined.

Previous studies indicated that ABV may feature sequence diversity in the region between the N and X genes [15], [18]. In a recent study, we detected persistent infection by ABV5 of an Eclectus parrot (*Eclectus roratus*), suffering from the feather picking disease [18]. Sequence analysis revealed that the upstream region of the X ORF of ABV5 is almost the same length as that of BDV, although the putative uORF of ABV5 is 9 nucleotides (nt) shorter than that of BDV (Figure 2A). In addition, we reported the identification of RBV N and X/P mRNA sequences in a cDNA library derived from a Bitis gabonica venom gland [12], [20]. Interestingly, in comparison with BDV, the sequence between the N and X ORFs of RBV appeared to have a 21 nt deletion (Figure 2A). These findings suggested that these genotypes may be useful tools for analysis of the functional interaction between X and P among bornavirus genotypes.

We aligned the amino acid sequences of X and P of BDV, ABV4, ABV5 and RBV. Consistent with phylogenetic analyses [9], [12], the X and P proteins of ABV5 showed a relatively high similarity with those of BDV (Figure 2B and C). In contrast, RBV seems to be distant from the other genotypes. The amino acid sequence identities and similarities among the genotypes are shown in Table 1. Previous studies revealed that the BDV X and P proteins contain several signal sequences, including nuclear localization (NLS) and nuclear export (NES) signals, putative binding sites for each other and phosphorylation sites 21–26. The BDV P protein has two proline-rich NLSs, located in the N- and C-terminal regions [23], [24]. A proline residue is substituted in the corresponding regions of ABV4 and 5 (Figure 2C). On the other hand, RBV has only one and three proline residues in the N- and C-terminal regions, respectively. In addition, the methionine-rich NES of the BDV P protein seems to be relatively highly conserved among the ABVs, but not in RBV (Figure 1C). The putative binding sites of the X and P proteins also seem to have evolved differently in RBV, compared to other genotypes (Figure 1B and C). These observations confirm the evolutionary diversity among bornavirus genotypes.

**Table 1:** Amino acid sequence homologies of X proteins and phosphoproteins among bornavirus genotypes.

P				
	BDV	ABV4	ABV5	RBV
BDV		63 (85)	76 (89)	41 (63)
ABV4	42 (55)		69 (87)	41 (65)
ABV5	58 (72)	55 (67)		43 (66)
RBV	34 (53)	38 (50)	34 (49)	
**X**				

The cells of the upper right and lower left sections show the calculated percentages of amino acid identity (similarity) of P and X proteins among bornavirus genotypes by NCBI BLAST, respectively.

Accession numbers used in this study are AB258389 (BDV strain huP2br), FJ169441 (ABV4), AB519144 (ABV5), BAI68158 (RBV N), AB714965 (RBV X), and AB714966 (RBV P).

### Conserved Interaction among Bornaviruses between the X and P Proteins

**Figure 3:**
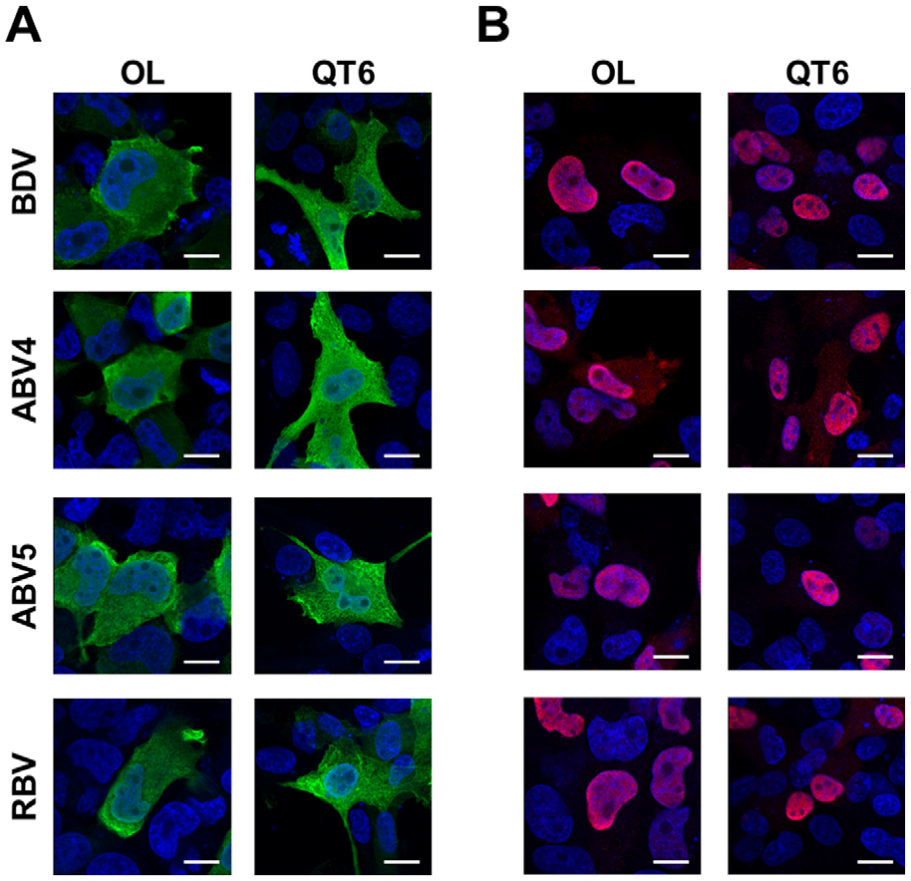
Intracellular localization of the bornavirus X and P proteins in mammalian and avian cell lines. Expression plasmids for the bornavirus X (A) and P (B) were transfected into OL and QT6 cells. Subcellular localizations of the recombinant proteins were detected by immunofluorescence assays using anti-Flag (A) (green) and -HA (B) (red) antibodies. Merged images with DAPI staining are shown. Scale bars are 10 µm.

**Figure 4:**
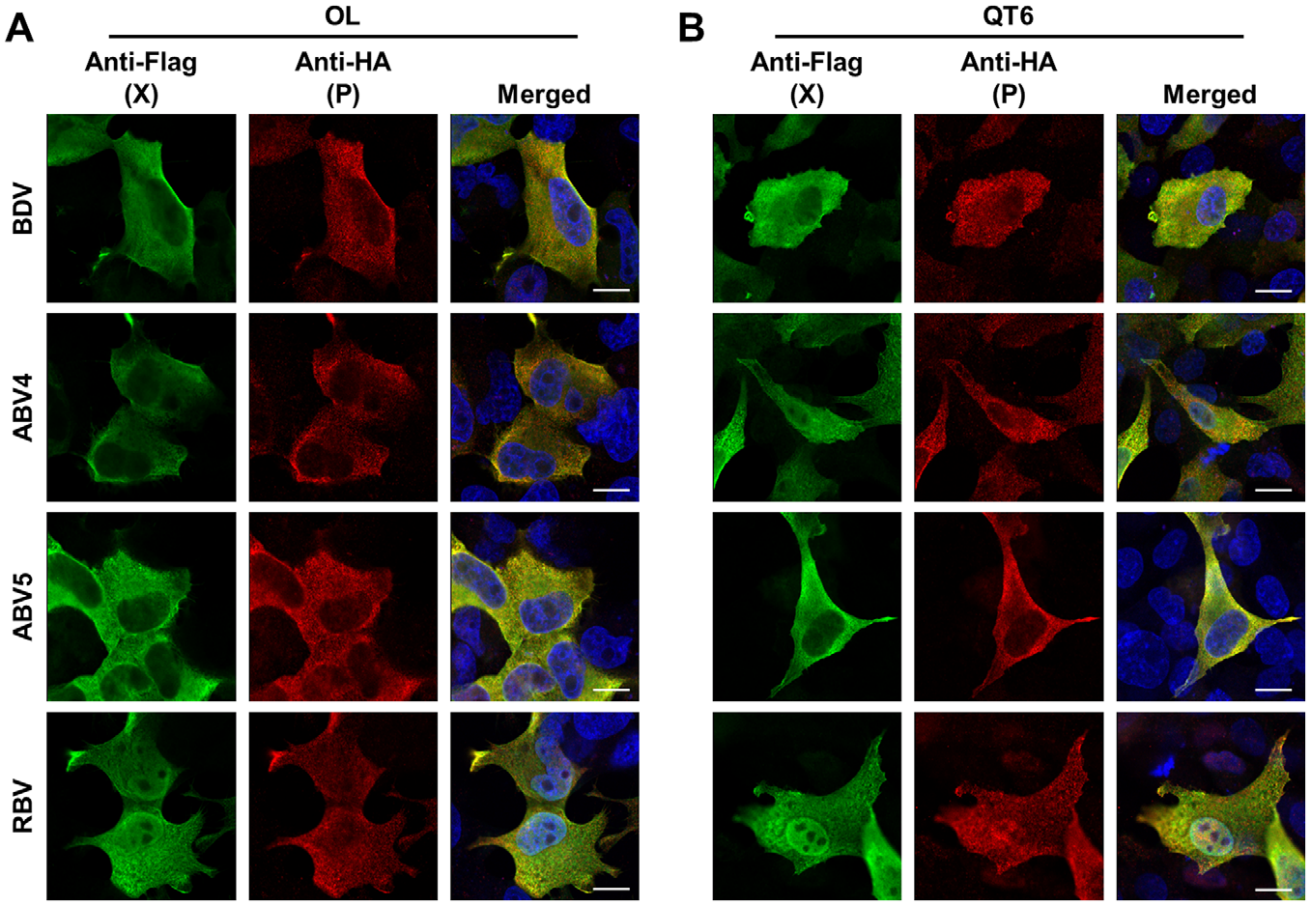
Nuclear export of P in the cells co-expressing the X protein. The X and P expression plasmids of each bornavirus genotype were co-transfected into OL (A) and QT6 (B) cell lines, and the intracellular distribution of the viral proteins was determined by immunofluorescence analysis. The recombinant X and P proteins were detected by anti-Flag (green) and -HA (red) antibodies, respectively. Merged images with DAPI staining are indicated. Scale bars are 10 µm.

To examine the functional conservation of the X and P proteins among bornavirus genotypes, we first determined the intracellular localization of the proteins. We transfected mammalian (OL) and avian (QT6) cell lines with Flag- and HA-tagged expression constructs of the X and P proteins, respectively, and detected their intranuclear distribution by immunofluorescence assays. As shown in Figure 3, the X and P proteins of ABV and RBV have a similar distribution, as seen for BDV, in both mammalian and avian cell lines. The X protein was distributed diffusely, and mainly located in the cytoplasm of the transfected cells (Figure 3A). On the other hand, a clear nuclear localization of the P protein was detected with all bornavirus genotypes (Figure 3B). Previous studies revealed that BDV P translocates from the nucleus to the cytoplasm with co-expression of the X protein [27]. To understand whether the P proteins of non-mammalian bornaviruses are also exported to the cytoplasm following interaction with their own partner, we co-transfected the X and P expression plasmids into OL and QT6 cells and examined the intracellular distribution of the P proteins at 24 h post-transfection. As shown in Figure 4, the expression of the X protein facilitated the cytoplasmic distribution of the P proteins of both ABV and RBV, as it does for BDV, although the P protein of RBV was seen to be retained in the nuclei to some extent.

**Figure 5:**
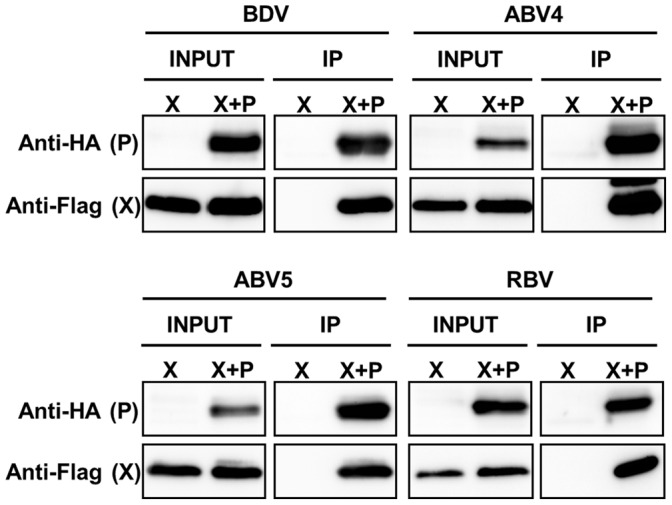
Intra-genotypic interaction between the X and P proteins of bornaviruses. Plasmids expressing the X and P proteins of each bornavirus genotype were co-transfected into 293T cells and interaction between X and P was detected by immunoprecipitation using anti-HA antibody. Immunoprecipitation of X was detected by anti-Flag antibody.

We also verified the interaction between the X and P proteins of non-mammalian bornaviruses. The X and P expression plasmids were co-transfected into 293T cells and the intra-genotypic interaction was examined by immunoprecipitation analysis. As shown in Figure 5, the X protein of non-mammalian bornaviruses was efficiently precipitated from the cell lysates with the P protein. All these results suggested that the function of the X protein as a regulator of the amount of intranuclear P in infected cells may be conserved evolutionarily.

### Inter-genotypic Interaction between the X and P Proteins of Vertebrate Bornaviruses

**Figure 6:**
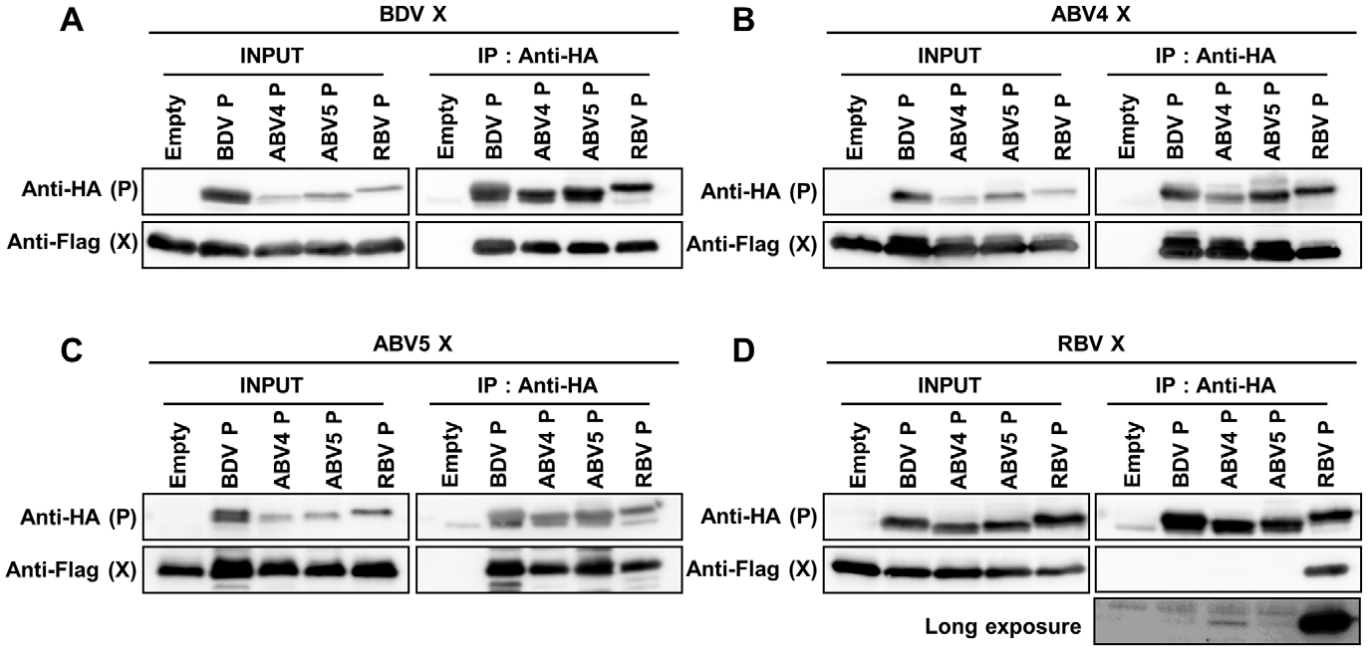
Inter-genotypic interaction between the bornavirus X and P proteins. Immunoprecipitation analysis was carried out using cells co-transfected with plasmids expressing Flag-tagged X proteins of BDV (A), ABV4 (B), ABV5 (C) or RBV (D) and HA-tagged P expression plasmids from each genotype indicated. After immunoprecipitation with anti-HA antibody, the precipitates were detected by anti-Flag antibody. A long exposure image of the membrane is shown for the inter-genotypic interaction of RBV X (D).

To understand the evolutionary relationship between bornavirus genotypes, we next examined the inter-genotypic interaction between the X and P proteins. 293T cells were transfected with the X and P expression plasmids with various combinations of the different genotypes and protein interactions were detected by immunoprecipitation analysis using anti-HA antibody. Interestingly, the P protein of all genotypes, including RBV, was shown to efficiently precipitate the X proteins of BDV and the ABVs (Figure 6A, B, C). In contrast, the inter-genotypic interaction of the RBV X protein was detected only in cells co-transfected with ABV4 P after a long exposure image of the membrane (Figure 6D). Although the weak interaction between RBV X and ABV4 P may be due to the over-expression experiment of the recombinant proteins, similar results were obtained in the experiment using immunofluorescence assay; the RBV P protein was translocated to the cytoplasm when the BDV and ABV X proteins were co-expressed in the cells, whereas the X protein of RBV was seemed to slightly affect the nuclear distribution of ABV4 P (Figure S1).

**Figure 7:**
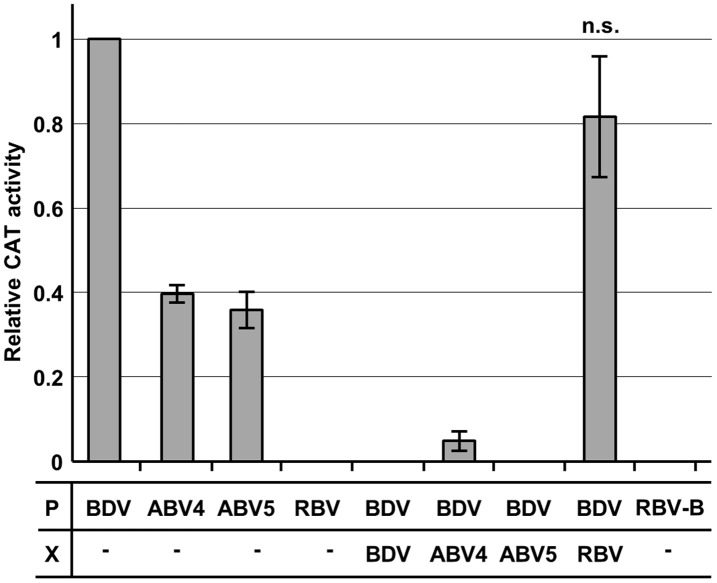
Compatible function of ABV X and P in a BDV minireplicon assay. BDV minireplicon assays were performed using the expression plasmids indicated, together with 0.125 ng of the minigenome construct and helper plasmids expressing BDV N (0.125 ng) and L (0.125 ng). The graph shows the mean ± SE of three independent experiments. At least three independent experiments were performed, except for RBV-B-transfection assay (n = 2). The differences were statistically significant (*P<*0.01, student t test), except for the assay using RBV X and RBV-BP. n.s., not significant.

We determined finally whether the proteins of non-mammalian bornaviruses can participate in the polymerase activity of BDV using a minireplicon system [28], which can reconstitute recombinant BDV nucleocapsids containing an artificial, genome-like reporter RNA (minigenome) following transfection of expression plasmids encoding BDV N, P, L, and the minigenome. Previous studies have demonstrated that BDV X can inhibit the viral polymerase activity in the minireplicon system [29]. We carried out the minireplicon assay using P expression plasmids of the non-mammalian bornaviruses instead of the BDV P plasmid. In addition, the assays were performed in the presence or absence of the X proteins from ABV and RBV. At 48 h post-transfection, BDV polymerase activity was determined by expression of chloramphenicol acetyltransferase (CAT), detected by ELISA. As shown in Figure 7, interestingly, the P proteins of ABV4 and 5, but not RBV, could initiate the polymerase activity of BDV up to 40% of the level of BDV P. A recombinant RBV P, which was added the five amino acid residues, Trp-Asp-Ile-Ile-Pro, important for the interaction with BDV N of BDV P (Figure 2C) [30] in the C-terminus, also failed to induce the CAT activity in the transfected cells (Figure 7, RBV-B). This result suggested that the lack of the activation of the minireplicon by RBV P might not be due to the limited interaction between RBV P and BDV N. In addition, the X proteins of ABVs also appeared to reduce markedly the polymerase activity of BDV, as does BDV X (Figure 7). On the other hand, as expected, the CAT activity did not decrease in cells co-expressing RBV X. Although species-specific host factors may be involved in the functional association of the viral proteins, our results suggested that RBV seems to have evolved distinctly from the other genotypes, in so far as the functional compatibility of the X and P proteins was lost.

## Discussion

In this study, we investigated the functional interaction of the X and P proteins of various bornaviruses to understand the evolutionary relationships among the genotypes. Sequence analysis revealed that the regions corresponding to the 5′ UTR of the putative X/P mRNA of ABV5 and RBV are classified into the BDV- and ABV2/4-type, respectively. The putative 5′ UTR of ABV5 was shown to be almost of the same length as that of mammalian bornavirus, whereas RBV contained an ABV2/4-type deletion in this region (Figure 2). In a previous study, we revealed using phylogenetic analysis that RBV may have branched before the separation of ABV and BDV [12], suggesting that additional sequences have been inserted into the 5′ UTR of the X/P mRNA of the BDV/ABV_CG_/ABV5 lineage during their evolution and after their divergence from the ABV2/4 lineage. This observation suggested that the acquired nucleotides in that lineage may have been involved in the adaptation to new host species or cellular environments. The fact that several host factors have been shown to bind to the 5′ UTR of BDV [16] may support this hypothesis.

Alignment of X and P protein sequences from various genotypes revealed that these are relatively well conserved between BDV and ABVs, but the sequence of RBV seems to differ significantly from those of the other genotypes, even in the signal sequences (Figure 2). This observation suggests that the proteins of RBV may have acquired distinct properties as a consequence of the evolution in a distinct host. In a previous study, we reported the C-terminal sequence of the N protein of RBV [12]. Interestingly, the C-terminal sequence of RBV N is well conserved with those of BDV and ABVs (Figure S2), suggesting that functional constraints on that region of the N proteins may have dictated the conservation of the sequence. It would be of interest to investigate whether a recombinant BDV N, with its C-terminal sequence derived from RBV N, can promote the polymerase activity of BDV.

By immunofluorescence analysis, using mammalian and avian cell lines, we showed that the X and P proteins of the non-mammalian bornaviruses tested exhibit a similar distribution to those of BDV in transfected cells (Figure 3) and that the co-expression of X with P induces efficient translocation of the P protein from the nucleus to the cytoplasm. The immunoprecipitation assay also confirmed the interaction between the X and P proteins of non-mammalian bornaviruses. These data strongly suggest that the functional interaction between the X and P proteins of bornaviruses has been conserved during their evolution and that control of the intranuclear level of the P protein may be a fundamental role of the bornavirus X protein. On the other hand, we also found that the nuclear export of the P by the X of RBV may not be optimal in the mammalian and avian cells (Figure 4). This observation suggested either that the RBV X may employ the different mechanism to transport the P to the cytoplasm from the nucleus, or that some reptile-specific host factors may be required for the proper function of the RBV X in the cells.

In a previous study, we showed that the 5′ UTR of the X/P mRNA of BDV contains elements that control the translation of the X protein [16]. We showed that interaction of the RNA helicase DDX21 with the predicted stem-loop structure in the 5′ UTR negatively regulates ribosomal initiation at the AUG codon of the X ORF. It was also shown that the P protein may enhance ribosomal reinitiation at the X ORF by inhibition of the interaction of DDX21 with the stem-loop structure, via interference with its phosphorylation [16]. Considering that the X proteins of non-mammalian bornaviruses seem to have a conserved function as regulatory proteins for maintenance of the optimal level of the P protein in the nucleus, the genotypes with a short 5′ UTR in the putative X/P mRNA may use a different mechanism to control the translation of the X protein in infected cells. Intriguingly, we found that, despite the short length of the 5′ UTR of the putative X/P mRNA in ABV4 and RBV, these seem to form stem-loop structures in a short stretch encompassing the 5′ UTR and the X encoding region (data not shown). This finding suggests the hypothesis that the stem-loop structures formed in the 5′ UTR of the X/P mRNA play a key role in the regulation of translation of the X protein of bornaviruses. Further studies are needed to verify the mechanism of regulation of translation of X from the X/P mRNA of the non-mammalian bornaviruses, via the predicted stem-loop structures of the various genotypes.

Our study also provided an interesting finding regarding the interaction between the X and P proteins. We showed that inter-genotypic interaction between X and P is found for all the genotypes, except for the X protein of RBV. Interestingly, although the sequence of the putative binding site between X and P in RBV appeared to be distinct from those in BDV and ABV, RBV P seemed to bind efficiently to the X proteins of both BDV and ABV and was translocated to the cytoplasm in the transfected cells. On the other hand, the X protein of RBV could not precipitate the P proteins of BDV and ABV5, and only a weak interaction was detected between RBV X and ABV4 P. This finding suggests that the functional constraints on the P protein during bornavirus evolution might have been stronger than those on the X protein. While the function of the P protein might be limited to acting as the polymerase of the viruses, the X protein may have been evolved to play several other roles in viral replication as previously reported [31]. In addition, considering that RBV X could not interact with the P protein of other genotypes, it is tempting to speculate that the X protein of RBV has evolved or has been evolving a specific function. The fact that the mitochondrial distribution of the X proteins is observed only in the BDV X-transfected cells (Figure S3) also support the hypothesis that the bornavirus X proteins of different genotypes may have been evolved differently. The co-evolutionary analysis of the X and P proteins of bornaviruses may provide interest insights into the genotype-specific evolution of the viral proteins.

In conclusion, we found the fundamental functions of the X and P proteins for the replication of bornaviruses may have been conserved during their evolution. Comparative study of the various genotypes of bornaviruses may be important for understanding not only the pathogenesis of the viruses but also the inter-species transmission of bornaviruses. Further studies using isolated viruses of different genotypes are needed to achieve a comprehensive analysis of the evolutionary relationships between the genotypes, as well as for the virological characterization of bornaviruses.

## Supporting Information

Figure S1 **Nuclear export of RBV and ABV P proteins by X proteins from different genotype.** Expression plasmids for the indicated bornavirus X and P proteins were transfected into OL cells. Subcellular localizations of the recombinant proteins were detected by immunofluorescence assay using anti-Flag (X: green) and -HA (P: red) antibodies. Merged images with DAPI staining are shown. Scale bars are 10 µm.
(TIF)

Figure S2 **Sequence comparison of the C-terminal regions of bornavirus N proteins.** Amino acids identical to the BDV sequence are indicated by dots. Gaps are indicated by dashes. Sequences identical in four and three genotypes are indicated by asterisks and dots, respectively, above the sequences.
(TIF)

Figure S3 **Subcellular localization of bornavirus X proteins.** Expression plasmids for the bornavirus X proteins were transfected into OL (A) and QT6 (B) cells. Forty-eight hours after the transfection, the cells were treated with MitoTracker Red CMXRos (Invitrogen) and fixed. Subcellular localizations of the recombinant proteins were detected by immunofluorescence assay using anti-Flag (X: green) antibody. Merged images with DAPI staining are shown. Scale bars are 10 µm.
(TIF)

## References

[1] Tomonaga K, Kobayashi T, Ikuta K (2002) Molecular and cellular biology of Borna disease virus infection. Microbes Infect 4: 491–500. 11932200 10.1016/s1286-4579(02)01564-2

[2] Gonzalez-Dunia D, Sauder C, de la Torre JC (1997) Borna disease virus and the brain. Brain Res Bull 44: 647–664. 9421127 10.1016/S0361-9230(97)00276-1 PMC7126547

[3] Rott R, Becht H (1995) Natural and experimental Borna disease in animals. Curr Top Microbiol Immunol 190: 17–30. 7789148 10.1007/978-3-642-78618-1_2

[4] Jordan I, Lipkin WI (2001) Borna disease virus. Rev Med Virol 11: 37–57. 11241801 10.1002/rmv.300 PMC7169201

[5] Schneider PA, Briese T, Zimmermann W, Ludwig H, Lipkin WI (1994) Sequence conservation in field and experimental isolates of Borna disease virus. J Virol 68: 63–68. 8254777 10.1128/jvi.68.1.63-68.1994 PMC236264

[6] Pleschka S, Staeheli P, Kolodziejek J, Richt JA, Nowotny N, et al (2001) Conservation of coding potential and terminal sequences in four different isolates of Borna disease virus. J Gen Virol 82: 2681–2690. 11602780 10.1099/0022-1317-82-11-2681

[7] Kolodziejek J, Durrwald R, Herzog S, Ehrensperger F, Lussy H, et al (2005) Genetic clustering of Borna disease virus natural animal isolates, laboratory and vaccine strains strongly reflects their regional geographical origin. J Gen Virol 86: 385–398. 15659758 10.1099/vir.0.80587-0

[8] Honkavuori KS, Shivaprasad HL, Williams BL, Quan PL, Hornig M, et al (2008) Novel Borna virus in psittacine birds with proventricular dilatation disease. Emerg Infect Dis 14: 1883–1886. 19046511 10.3201/eid1412.080984 PMC2634650

[9] Kistler AL, Gancz A, Clubb S, Skewes-Cox P, Fischer K, et al (2008) Recovery of divergent avian bornaviruses from cases of proventricular dilatation disease: Identification of a candidate etiologic agent. Virol J 5: 88. 18671869 10.1186/1743-422X-5-88 PMC2546392

[10] Weissenbock H, Sekulin K, Bakonyi T, Hogler S, Nowotny N (2009) Novel avian bornavirus in a nonpsittacine species (canary; *Serinus canaria*) with enteric ganglioneuritis and encephalitis. J Virol 83: 11367–11371. 19706702 10.1128/JVI.01343-09 PMC2772780

[11] Delnatte P, Berkvens C, Kummrow M, Smith DA, Campbell D, et al (2011) New genotype of avian bornavirus in wild geese and trumpeter swans in Canada. Vet Rec 169: 108. 10.1136/vr.d4620 21784813

[12] Horie M, Honda T, Suzuki Y, Kobayashi Y, Daito T, et al (2010) Endogenous non-retroviral RNA virus elements in mammalian genomes. Nature 463: 84–87. 20054395 10.1038/nature08695 PMC2818285

[13] Weissenbock H, Bakonyi T, Sekulin K, Ehrensperger F, Doneley RJ, et al (2009) Avian bornaviruses in psittacine birds from Europe and Australia with proventricular dilatation disease. Emerg Infect Dis 15: 1453–1459. 19788814 10.3201/eid1509.090353 PMC2819881

[14] Rubbenstroth D, Rinder M, Kaspers B, Staeheli P (2012) Efficient isolation of avian bornaviruses (ABV) from naturally infected psittacine birds and identification of a new ABV genotype from a salmon-crested cockatoo (*Cacatua moluccensis*). Vet Microbiol. [Epub ahead of print]. 10.1016/j.vetmic.2012.07.004 22824256

[15] Payne S, Covaleda L, Jianhua G, Swafford S, Baroch J, et al (2011) Detection and characterization of a distinct bornavirus lineage from healthy Canada geese (*Branta canadensis*). J Virol 85: 12053–12056. 21900161 10.1128/JVI.05700-11 PMC3209299

[16] Watanabe Y, Ohtaki N, Hayashi Y, Ikuta K, Tomonaga K (2009) Autogenous translational regulation of the Borna disease virus negative control factor X from polycistronic mRNA using host RNA helicases. PLoS Pathog 5: e1000654. 19893625 10.1371/journal.ppat.1000654 PMC2766071

[17] Poenisch M, Staeheli P, Schneider U (2008) Viral accessory protein X stimulates the assembly of functional Borna disease virus polymerase complexes. J Gen Virol 89: 1442–1445. 18474560 10.1099/vir.0.2008/000638-0

[18] Horie M, Ueda K, Ueda A, Honda T, Tomonaga K (2012) Detection of avian bornavirus 5 RNA in eclectus roratus with feather picking disorder. Microbiol Immunol 56: 346–349. 22309239 10.1111/j.1348-0421.2012.00436.x

[19] Nakamura Y, Takahashi H, Shoya Y, Nakaya T, Watanabe M, et al (2000) Isolation of Borna disease virus from human brain tissue. J Virol 74: 4601–4611. 10775596 10.1128/jvi.74.10.4601-4611.2000 PMC111980

[20] Francischetti IM, My-Pham V, Harrison J, Garfield MK, Ribeiro JM (2004) Bitis gabonica (*Gaboon viper*) snake venom gland: Toward a catalog for the full-length transcripts (cDNA) and proteins. Gene 337: 55–69. 15276202 10.1016/j.gene.2004.03.024 PMC2907531

[21] Schwemmle M, Salvatore M, Shi L, Richt J, Lee CH, et al (1998) Interactions of the Borna disease virus P, N, and X proteins and their functional implications. J Biol Chem 273: 9007–9012. 9535888 10.1074/jbc.273.15.9007

[22] Malik TH, Kishi M, Lai PK (2000) Characterization of the P protein-binding domain on the 10-kilodalton protein of Borna disease virus. J Virol 74: 3413–3417. 10708460 10.1128/jvi.74.7.3413-3417.2000 PMC111844

[23] Schwemmle M, Jehle C, Shoemaker T, Lipkin WI (1999) Characterization of the major nuclear localization signal of the Borna disease virus phosphoprotein. J Gen Virol 80: 97–100. 9934690 10.1099/0022-1317-80-1-97

[24] Shoya Y, Kobayashi T, Koda T, Ikuta K, Kakinuma M, et al (1998) Two proline-rich nuclear localization signals in the amino- and carboxyl-terminal regions of the Borna disease virus phosphoprotein. J Virol 72: 9755–9762. 9811710 10.1128/jvi.72.12.9755-9762.1998 PMC110486

[25] Yanai H, Kobayashi T, Hayashi Y, Watanabe Y, Ohtaki N, et al (2006) A methionine-rich domain mediates CRM1-dependent nuclear export activity of Borna disease virus phosphoprotein. J Virol 80: 1121–1129. 16414989 10.1128/JVI.80.3.1121-1129.2006 PMC1346931

[26] Wolff T, Unterstab G, Heins G, Richt JA, Kann M (2002) Characterization of an unusual importin alpha binding motif in the Borna disease virus p10 protein that directs nuclear import. J Biol Chem 277: 12151–12157. 11796712 10.1074/jbc.M109103200

[27] Kobayashi T, Zhang G, Lee BJ, Baba S, Yamashita M, et al (2003) Modulation of Borna disease virus phosphoprotein nuclear localization by the viral protein X encoded in the overlapping open reading frame. J Virol 77: 8099–8107. 12829848 10.1128/JVI.77.14.8099-8107.2003 PMC161951

[28] Yanai H, Hayashi Y, Watanabe Y, Ohtaki N, Kobayashi T, et al (2006) Development of a novel Borna disease virus reverse genetics system using RNA polymerase II promoter and SV40 nuclear import signal. Microbes Infect 8: 1522–1529. 16697679 10.1016/j.micinf.2006.01.010

[29] Schneider U, Naegele M, Staeheli P, Schwemmle M (2003) Active Borna disease virus polymerase complex requires a distinct nucleoprotein-to-phosphoprotein ratio but no viral X protein. J Virol 77: 11781–11789. 14557662 10.1128/JVI.77.21.11781-11789.2003 PMC229352

[30] Schwemmle M, Salvatore M, Shi L, Richt J, Lee CH, et al (1998) Interactions of the Borna disease virus P, N, and X proteins and their functional implications. J Biol Chem 273: 9007–9012. 9535888 10.1074/jbc.273.15.9007

[31] Poenisch M, Burger N, Staeheli P, Bauer G, Schneider U (2009) Protein X of Borna disease virus inhibits apoptosis and promotes viral persistence in the central nervous systems of newborn-infected rats. J Virol 83: 4297–4307. 19211764 10.1128/JVI.02321-08 PMC2668499

