# Supplementary figures and images for: Evolutionarily Conserved Interaction between the Phosphoproteins and X Proteins of Bornaviruses from Different Vertebrate Species
Source: PLoS One. 2012 Dec 7;7(12):e51161. doi: 10.1371/journal.pone.0051161 (PMC3517445; doi:10.1371/journal.pone.0051161)

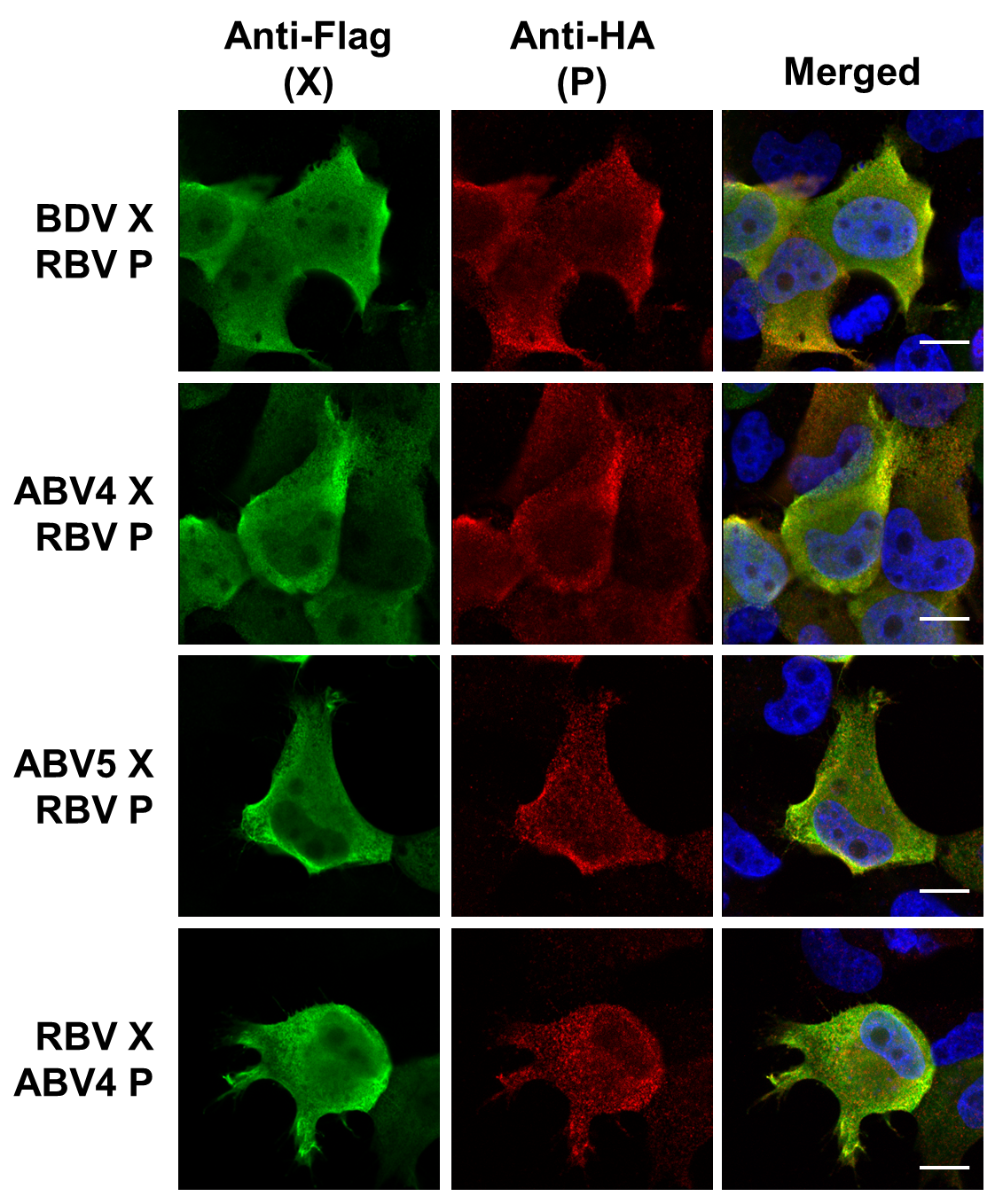

Supplement: Figure S1 — Nuclear export of RBV and ABV P proteins by X proteins from different genotype. Expression plasmids for the indicated bornavirus X and P proteins were transfected into OL cells. Subcellular localizations of the recombinant proteins were detected by immunofluorescence assay using anti-Flag (X: green) and -HA (P: red) antibodies. Merged images with DAPI staining are shown. Scale bars are 10 µm. (TIF) [file pone.0051161.s001.tif]

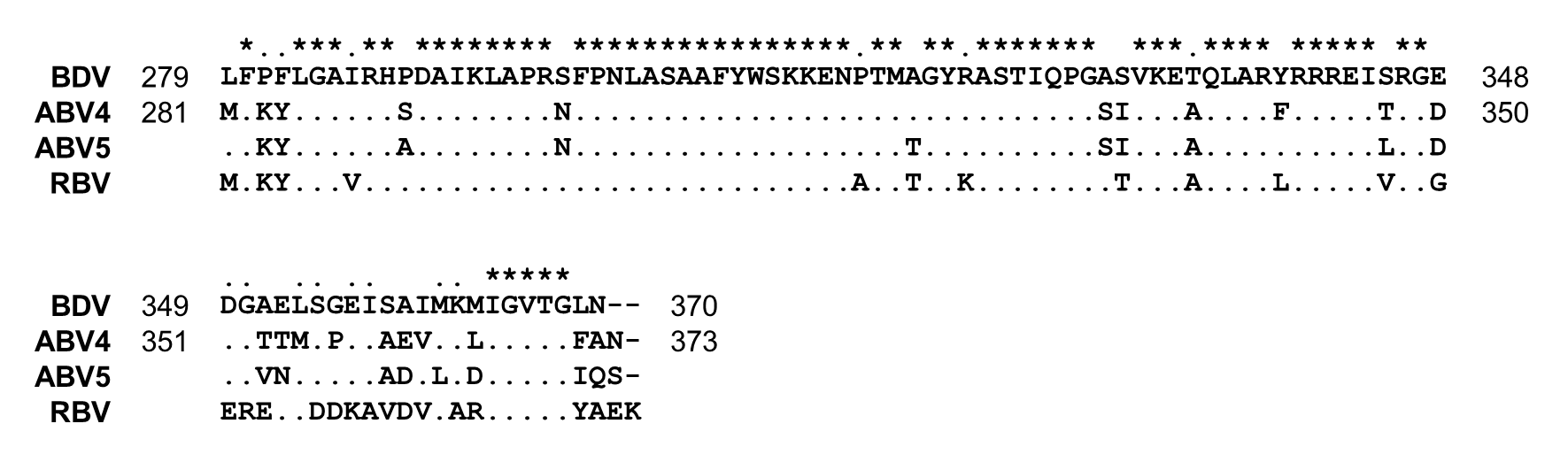

Supplement: Figure S2 — Sequence comparison of the C-terminal regions of bornavirus N proteins. Amino acids identical to the BDV sequence are indicated by dots. Gaps are indicated by dashes. Sequences identical in four and three genotypes are indicated by asterisks and dots, respectively, above the sequences. (TIF) [file pone.0051161.s002.tif]

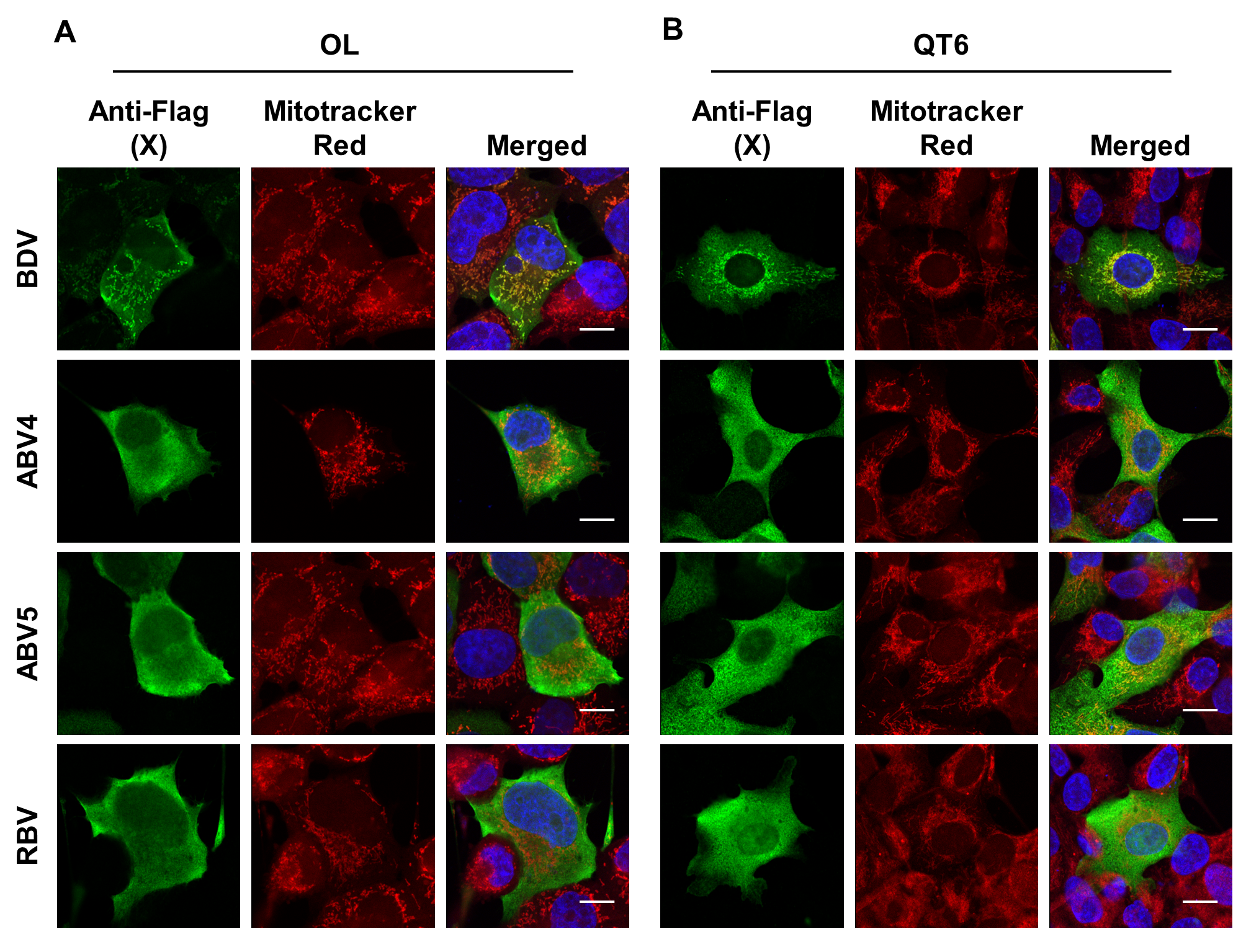

Supplement: Figure S3 — Subcellular localization of bornavirus X proteins. Expression plasmids for the bornavirus X proteins were transfected into OL (A) and QT6 (B) cells. Forty-eight hours after the transfection, the cells were treated with MitoTracker Red CMXRos (Invitrogen) and fixed. Subcellular localizations of the recombinant proteins were detected by immunofluorescence assay using anti-Flag (X: green) antibody. Merged images with DAPI staining are shown. Scale bars are 10 µm. (TIF) [file pone.0051161.s003.tif]
